# COVID-19, normative attitudes and pluralistic ignorance in employer-employee relationships

**DOI:** 10.1186/s12651-022-00325-4

**Published:** 2022-11-13

**Authors:** Martin Abraham, Matthias Collischon, Veronika Grimm, Frauke Kreuter, Klaus Moser, Cornelia Niessen, Claus Schnabel, Gesine Stephan, Mark Trappmann, Tobias Wolbring

**Affiliations:** 1 School of Business, Economics and Society, Friedrich-Alexander-Universität Erlangen-Nürnberg, Findelgasse 7, 90402 Nürnberg, Germany; 2 Institute for Employment Research, Nürnberg, Germany; 3 Department of Statistics, Ludwig-Maximilians-Universität München, Munich, Germany; 4 Department of Psychology, Friedrich-Alexander-Universität Erlangen-Nürnberg, Nürnberg, Germany

**Keywords:** Employment relationship, Social norms, Pluralistic ignorance, Short-time work, Working from home, D91, J50, J81

## Abstract

Employment relationships are embedded in a network of social norms that provide an implicit framework for desired behaviour, especially if contractual solutions are weak. The COVID-19 pandemic has brought about major changes that have led to situations, such as the scope of short-time work or home-based work in a firm. Against this backdrop, our study addresses three questions: first, are there social norms dealing with these changes; second, are there differences in attitudes between employees and supervisors (misalignment); and third, are there differences between respondents’ average attitudes and the attitudes expected to exist in the population (pluralistic ignorance). We find that for the assignment of short-time work and of work at home, there are shared normative attitudes with only small differences between supervisors and nonsupervisors. Moreover, there is evidence for pluralistic ignorance; asked for the perceived opinion of others, respondents over- or underestimated the consensus in the (survey) population. Such pluralistic ignorance can contribute to the upholding of a norm even if individuals do not support the norm, with potentially far-reaching consequences for the quality of the employment relationship and the functioning of the organization. Our results show that, especially in times of change, social norms should be considered for the analysis of labour markets.

## Introduction

Employment relationships are not only economic exchange relationships, they are also embedded in society and thus shaped by the normative attitudes of employees, employers and other actors (Granovetter 1985). These attitudes encompass, among others, concerns about fairness, reciprocity or authority, and they influence behaviour beyond the direct costs/benefit of an exchange relationship (Akerlof 1982; Fehr et al. 1998). At the aggregate level, normative attitudes manifest themselves in social norms (Bicchieri 2006) and shared ideas about how to behave, which people feel obliged to obey (Bicchieri 2006, 2017). Usually, social norms are enforced by means of positive or negative sanctions such as social recognition or disapproval (Posner and Rasmusen 1999). However, the internalization of social norms can also create incentives for norm compliance, as the violation of internalized norms can lead to negative feelings, such as shame, regret and a bad conscience (Maibom 2010).

Labour market research has neglected this normative embeddedness of employment relationships to some extent. Experimental studies have clearly documented the power of social norms such as fairness and reciprocity for human behaviour in general (e.g., Fehr and Schmidt 2006) and have also demonstrated their relevance in labour market contexts (e.g., Charness 2004). There is, however, little empirical research on how such general normative principles translate into more specific, local normative expectations regarding behaviour in the workplace. Likewise, little is known about how they change over time. In times of an economic or social crisis, when social norms often adjust to changing circumstances, existing conflicts may intensify or new conflicts might arise. For example, the COVID-19 crisis has very likely led to a temporary or even permanent rearrangement of social norms concerning core topics at the workplace, such as working at home, short-time work, and the sharing of private information. Based on the general theoretical argument that social norms can contribute to solve collective problems (Coleman 1990; North 1991), it can be argued that the pandemic led to a specific demand for social norms at the workplace. However, the pandemic also raised tensions between different interests, such as maintaining productive workflows, ensuring employees’ health, and considering private responsibilities. Thus it is unclear whether norms supporting the interests of one group will be rejected by those who have to bear the costs for this solution.

Taking advantage of this rather rapid shift in attention towards workplace-related norms, our study examines normative attitudes towards a selected set of behaviours that received particular public attention in the context of the COVID-19 pandemic. To our knowledge, this is the first study to address this topic. Specifically, we look at attitudes towards the assignment and compensation of short-time work, the opportunity to work from home, and the sharing of private information on employees’ vacation destination with the employer. All four examples are specifically interesting because they are dealing with behaviour that has the potential to buffer the effects of the pandemic on employees and/or employers. At the same time, the four norms address conflicting interests since the behaviour imposes either costs on the employer or the employee (by reducing privacy). Due to these conflicting interests of employers and employees, these examples enable us to investigate if those asymmetric interests lead to different normative attitudes. Therefore, we distinguish between employees with and without supervisory functions, assuming that the latter group represents the interests of employers, at least to a certain extent.

First, we ask whether there are consistent patterns for a majority of respondents, indicating that there exist social norms on this subject (RQ1). Second, we explore whether and to what extent there is a misalignment of normative attitudes between employees and supervisors (RQ2). If supervisors support a norm less often than employees do, this is a source of potential conflict in the employment relationship (Görges and Nosenzo 2020). Third, we look at differences between respondents’ average attitudes and the attitudes they expect to exist in the population (RQ3). This will allow us to identify whether and to what degree people overestimate support for a social norm. This is important because under such circumstances, people may contribute to the upholding of the norm even if they do not support the norm itself. This behaviour is also known as pluralistic ignorance (Shamir and Shamir 1997). We examine whether and to what degree such pluralistic ignorance occurs in the context of the COVID-19 pandemic. By answering these questions, we shed light on the role of norms for employment relationships. Norms guide what is adequate behaviour and, thus, have the potential to regulate conflicts. However, this requires that not only a sufficient number of people support a norm (RQ1), but that the relevant type of actors also agree on the norm (RQ2). Finally, if individuals overestimate the support of a norm (RQ3), this can help to uphold the norm in the short run, but may lead to problems in the long run in case people learn about that fact.

Based on the empirical results from a large-scale, representative sample of labour market participants in Germany, we show that most respondents hold similar normative attitudes regarding the obligations of employers and employees in times of the COVID-19 crisis. Depending on the specific question (see Appendix  for the original questions), 60 to 80 percent of the respondents agree or somewhat agree with the following statements: i) Companies should avoid introducing short-term work as long as their financial resources allow them to avoid doing so; ii) If they utilize short-time work, they should top up the short-time allowance that employees receive from unemployment insurance; iii) Employers should give employees (especially those with children) the opportunity to work from home even if not all tasks can be completed there; and iv) Employees should disclose the location of their last vacation to their employer during the COVID-19 pandemic. Moreover, regarding short-time work and private information about the last vacation, the results suggest that there is a tendency for pluralistic ignorance in favour of employee rights. Many respondents, also supervisors, seem to overestimate the extent of agreement in the population with attitudes that strengthen the position of the employee. These results support the idea that employment relationships are regulated by social norms. This regulation is particularly important in times of crisis since it reduces tensions, conflicts and insecurities. The next section provides a short review of the literature dealing with the role of norms in employment relationships, which is the basis for our theoretical and empirical analysis.

## Norms in employment relationships

The exchange relationship between employers and employees is one of the most important economic transactions in modern societies. How these employment relationships (ERs) function has large effects on the living conditions of employees, the performance of firms, and the functioning of the economic system and, thus, the welfare of a society. This importance is mirrored by the fact that ERs are highly regulated by institutions such as labour law. However, at a second glance, this formal regulation often provides only general guard rails and formal procedures to handle conflicts. Beyond these institutional guidelines and constraints, ERs are characterized by a high degree of informal agreements, leeway, and bargaining. This has led to the general question in research on labour markets and organizational behaviour of how these ERs should be designed to maximize welfare for employers, employees, and society.

In light of this question, research has led to a multitude of theoretical approaches, which are based, among others, on the proper design of incentives, organizational structures and individual expectations and commitments. However, during recent decades, it has become clear from research in sociology, economics, and psychology that this classical economic literature partly neglects the role of social norms in explanations of the way ERs function. Following the concept of Bicchieri and others (Bicchieri 2006, 2017; Cialdini and Trost 1998), a social norm is a rule about how to behave, which is shared by a sufficiently large number of individuals in a population, and a deviation from the norm leads to a sanction with a positive probability. In a most general sense, social norms are rules for behaviour in a given population. We call the related constructs at the individual level normative attitudes. At the core of social norms is the expectation that others will evaluate an individual’s behaviour based on their normative attitudes and that this evaluation may have consequences. In contrast to formal norms such as laws, the enforcement of social norms does not rely on institutionalized mechanisms. Instead, social norms are typically enforced by the groups and networks in which an actor is embedded or they work through internalization (Horne and Mollborn 2020).

Although there is a broadly shared understanding that norms are a basic element of societies (Coleman 1990; Ellickson 2001; Hechter and Opp 2001; North 1991), the explicit analysis of norms regarding ER is scarce, fragmented along disciplinary boundaries, and spread across different strands of the literature (Horne and Mollborn 2020). There is a small body of literature on matching and contracting that shows that social norms influence the process of applying and hiring for jobs (e.g., Akerlof and Kranton 2000; Barr et al. 2018; Crandall et al. 2002; Hurley-Hanson and Giannantonio 2006; Mack and Rainey 1990; Seiter and Sandry 2003; Stewart et al. 2008). A set of studies has found that normative attitudes on fairness and justice are important determinants for the assessment of layoffs (Charness and Levine 2000; Engelstad 1997; Gerlach et al. 2008; Pfeifer 2007; Struck et al. 2008). Another set of studies examines the role of norms for the relationship between employer and employee, with the primary focus on wages and compensation as the most prominent example. It has been shown that norms influence employees’ reactions to wages, compensation and working conditions (Breza et al. 2018; Cohn et al. 2014; Gerlach et al. 2008; Kaur 2019; Rost and Weibel 2013). Beyond fair payment, only a limited number of studies explicitly address the role of social norms on other workplace dimensions. Exceptions are studies on the role of social norms on promotions (Beehr and Taber 1993; Lashbrook 1996) or on the flexibility of work schedules, where the norm of standard work hours may lead to a penalty for those working less (Coltrane et al. 2013; Epstein et al. 1999; Rudman and Mescher 2013).

In sum, social norms in organizations are often acknowledged in theory (e.g., Ajzen 1991) but rarely explicitly measured or examined as an important part of ERs (Hammer et al. 2004). Specifically, there is a considerable lack of knowledge about which specific norms exist and to what extent the relevant actors—employers and employees—acknowledge and adhere to this norm. With this paper, we contribute in a first step to overcoming this deficit by describing the dispersion of specific norms that became especially relevant in the COVID pandemic. These are injunctive norms prescribing behaviour for directing short-time work, granting work at home, and disclosing private behaviour, here the example of the vacation location of the employee.

Our first focus is primarily explorative: Is there a clear majority who supports the respective norm? Moreover, we look into differences between these norms. People are expected to support a norm more the greater the norm addresses their (financial or other) needs. As a second research goal, we focus on differences between employees with and without supervisory functions. To abstain from short-time work and to offer work from home usually leads to costs for the employer and should also affect how supervisors as agents of the employer perceive related norms. Based on the assumption that people tend to reject norms more if they lead to disadvantages, this leads to the hypothesis that supervisors support a norm favouring employees (short-time work, work from home) less than standard employees do. This mismatch is called misalignment of norms between employer and employee (Görges and Nosenzo 2020) and may result in tensions and conflicts since one side of the ER expects a certain behaviour more strongly than the other side. Moreover, since employees are usually considered more in need of protection than employers and imbalanced power relationships trigger norms (Stolte 1987), norms addressing employee needs should be more widely accepted than those addressing the needs of employers.

In a third step, we examine the extent of an information bias regarding the norm’s dispersion in the population. As has already been mentioned, individuals may have a misguided assessment of the average attitudes, beliefs, and behaviours of others. In this case, the perceived norm differs from the actual norm that is present in the group investigated. If members in a social group jointly overestimate the extent to which all others believe in a given norm, the group supports the norm, although only a minority hold attitudes in accordance with the norm. This phenomenon is also known as pluralistic ignorance (Allport 1924; Shamir and Shamir 1997). It has been observed for various normative behaviours, such as the consumption of alcohol (Prentice and Miller 1993), opinions on foreign policy (Todorov and Mandisodza 2004) and the formation of romantic relationships (Vorauer and Ratner 1996). Specifically, for ERs, Munsch et al. (2014) and Miyajima and Yamaguchi (2017) investigate pluralistic ignorance regarding the assessment of flexible working arrangements. Munsch et al. (2014) experimentally test the hypothesis that individuals believe others view flexworkers less positively than they do. The authors confirm this hypothesis on the basis of two vignette studies among convenience samples of M-Turk workers. Moreover, they assess whether this bias can be reduced by providing information on organizational leaders’ engagement in flexible work. The results show that bias against flextime (but not flexplace) workers was attenuated when the majority of high-status employees worked flexibly. Similarly, Miyajima and Yamaguchi (2017) examined pluralistic ignorance among men about the possibility of taking paternity leave in Japan. They find that male employees overestimate other men's negative attitudes towards paternity leave. Moreover, among those with positive attitudes towards taking leave, the belief that others have negative attitudes led to a reduced willingness to take paternity leave.

For this third part of our study, we explore whether we can find indications of pluralistic ignorance for the five normative attitudes we focus on in our empirical analysis. Theoretically, it can be assumed that more communication on the subject of a norm may reduce the tendency for pluralistic ignorance in a population (Bicchieri 2016; Munsch et al. 2014). Since the pandemic intensified the need for regulation (such as employee protection against layoffs) or created new problems (such as the employer’s interest in the location of employees’ vacations to assess the risk of infection for other staff members), we assume that people’s attitudes or their beliefs about the attitudes of others may have shifted. These changes, however, can result in pluralistic ignorance, since people may over- or underestimate the extent to which others support a norm. Consequently, as a second hypothesis, we expect that our four normative attitudes related to short-time work, the right to work from home with and without children, respectively, and the employer’s aspiration for information on the employee’s vacation, are potential candidates for pluralistic ignorance. Moreover, we expect that pluralistic ignorance should be smaller for topics that received more public attention during the pandemic than for those topics that have been less publicly discussed.

## Pandemic-related and institutional background

Our analysis focuses on the German labour market during the pandemic. As in many other countries, the first infection in Germany was recorded at the end of January 2020. The government introduced the first extensive restrictions in March 2020. When possible, employees were advised to work from home. During summer 2020, the situation somewhat improved, and restrictions were relaxed, but this was followed by a long period of increases in infection rates until October 2020, when caseloads increased significantly. During November 2020 and December 2020, Germany again tightened COVID-19 restrictions, which were still in place when we conducted our survey at the beginning of 2021.

The COVID-19 pandemic put severe pressure on the German labour market. The situation was especially difficult for the service sector, such as gastronomy, but manufacturing also suffered from disrupted supply chains and a shortage of upstream products. However, labour demand in Germany was comparably robust during the pandemic (Gartner et al. 2021). This can be explained by an intensive labour market policy targeted at avoiding layoffs and job losses (see Bonin et al. 2021 for an overview). Especially successful was the extensive application of short-time allowances (Pusch and Seifert 2021). A firm could register for short-time work if at least ten percent of its employees were affected by a work loss of more than ten percent. For those employees affected by the short-time allowance, the unemployment insurance paid a certain share of the wage. This share varied between 60 and 87 percent during the pandemic (depending on whether there were children in the household and the duration of short-time work) and prevented layoffs. It also, however, led to a loss of income for the respective workers in the firm.

Whereas short-term allowances have been applied for a long time and in different crises (Möller 2010), the question of who could work from home was comparably new. Although working from home seemed to be an effective measure during the pandemic, there was a debate about its assignment. This finally resulted in employers’ temporary duty to offer work from home as long as there were no compelling operational reasons to the contrary *(§4 Arbeitsschutzverordnung*), issued in January 2021. This led to a surge in employees working from home, albeit mostly white-collar workers.

Finally, when vacation trips were possible again after the first wave of the pandemic, there was a short discussion about the question of whether employers have the right to know where their employees went for vacation. Contrary to working at home, this issue was not solved by a new law, and it is still unclear in which cases an employee is required to give this information to the employer. According to the prevailing opinion among labour law experts, employees are obliged to provide information as to whether the vacation took place in a high-risk area. However, the employer has to verify that this information is necessary to protect other staff members.

Of the three topics—short-time work, the right to work from home, and the employer’s desire for information on the employee’s vacation—working from home was the most intensely discussed. A first indication for this is provided by an evaluation of Google Trends data, where the topic “home office & corona” showed two exceptional peeks when looking at the search quest of Google users (see Appendix). This corresponds with the fact that a legal right to work from home was not only highly discussed but also passed in parliament (Corona Datenplattform 2021). Consequently, we assume that the informational exchange on this topic among friends and colleagues was much higher than for the other two topics.

## Data and operationalization

We use data from the High-Frequency Online Personal Panel (HOPP) (see Haas et al. 2021; Volkert et al. 2021) of the Institute for Employment Research (IAB). The survey is based on a random sample of individuals drawn from the Integrated Employment Biographies (IEB) (see Antoni et al. 2019). More specifically, only individuals who had at least one IEB spell during the year 2018 were sampled. The IEB covers all times in one of the following states: i) employment (except self-employment and civil servants), ii) unemployment or job search, (iii) unemployment benefit receipt, (iv) welfare benefit receipt, and (v) participation in labour market programmes.

A total of 200,000 individuals were contacted by mail in May 2020 and asked to participate in the online panel. During the first wave, the response rate amounted to 5.7% (11,331 participants). Over time, the number of respondents declined due to panel attrition, and in wave 5, a refreshment sample was invited to participate (100,000 persons, again by mail). The data we use stem from wave 7 of HOPP, which was conducted during January/February 2021. A total of 6,344 persons participated in this wave, and 5,836 gave consent to link their answers to their administrative records.^1^ Calibration weights adjusting the respondents of each wave to known distributions on a rich set of variables from the administrative data they were sampled from are available (compare Volkert et al. 2021). In the following, all our analyses are based on the weighted sample.

We introduced specific questions on normative attitudes regarding employment relationships during the COVID-19 pandemic into this seventh wave. Each response scale ranged from “strongly agree” (1) to “strongly disagree” (4). We first asked for the respondents’ own attitudes on topics related to ERs that were at the centre of attention during the COVID-19 pandemic (which is often called the corona(virus) crisis in Germany). The question was as follows (for the original German version, see Appendix):

*Now it’s about how employers are dealing with the aftermath of the Corona crisis: To what extent do you personally agree with the following statements*?

- *Employers have a moral duty to avoid short-term work for their employees as long as there are still financial reserves in the company.*
- *Employers have a moral duty to top up the short-time allowance as long as there are still financial reserves in the company.*
- *Employees without children should be able to work at home even if they cannot perform all tasks there.*
- *Employees with children should be able to work at home even if they cannot complete all tasks there.*
- *Employees have a moral duty during the corona crisis to tell their employer where they have been on vacation.*

Afterwards, for the same items, we asked for the assessment of the majority attitude, among others:

*Your own opinion does not always correspond to that of the majority. What do you think the majority of working people will tick for the following statements regarding how an employer or an employee is dealing with the consequences of the corona crisis?*

Furthermore, the survey asked participants the questions *“Do you have a supervisory function in the company?”* and *“How many people do you directly or indirectly supervise?”*. We hypothesized that employees with a supervisory function locate themselves closer to the employer than those without such a function. Table 1 shows the variables used for our analysis and the respective sample descriptives. Information on education, experience, tenure, the number of employees at one’s establishment, citizenship and daily pay was obtained from administrative records.^2^ For the following analysis, we include all individuals who answered the questions on normative attitudes as well as control variables in the analysis. The analysis sample amounts to 4,609 observations and only includes individuals who are currently employed.

**Table 1:** Sample descriptives (weighted)

	All		Nonsupervisors		Supervisors	
	Mean	Std. dev.	Mean	Std. dev.	Mean	Std. dev.
Female	0.48	0.50	0.51	0.50	0.36	0.48
Age	43.70	12.40	43.26	12.66	45.75	10.92
Child under 18 in hh (0/1)	0.36	0.48	0.35	0.48	0.39	0.49
Short-time allowance (0/1)	0.10	0.30	0.10	0.30	0.11	0.31
Possibility for working at home (0/1)	0.43	0.50	0.41	0.49	0.54	0.50
Education: No vocational Training	0.09	0.28	0.10	0.30	0.05	0.21
Education: Vocational training	0.50	0.50	0.50	0.50	0.48	0.50
Education: Upper Secondary	0.05	0.21	0.05	0.22	0.04	0.20
Education: Upper Secondary + voc training	0.15	0.36	0.15	0.36	0.15	0.36
Education: University or FH	0.22	0.41	0.20	0.40	0.28	0.45
Experience (yrs)	17.91	10.89	17.29	10.94	20.73	10.21
Tenure (yrs)	5.21	6.59	4.78	6.38	7.18	7.20
No. of employees in establishment (2019)	925.21	3679.13	935.20	3797.41	879.02	3075.38
Daily pay in € (2019)	100.09	63.41	91.82	60.54	138.32	62.35
Foreign citizen (0/1)	0.08	0.26	0.08	0.27	0.07	0.26
Observations	4609		3599		1010	

Source: HOPP, wave 7

## Empirical results

Table 2 shows the descriptive results for our five questions on normative attitudes. For the odd-numbered columns, we show the means for our four-point answer scale. In the even-numbered columns, we collapsed the four-point answering scale to a binary measure by merging the two positive items “agree” and “strongly agree” and the two negative items “disagree” and “strongly disagree”. Displayed are the percentages for the “strongly agree” items. The first two columns show the full sample, the third and fourth columns show employees without any supervisory function, and the fifth and sixth columns show those for supervisors.

**Table 2:** Agreement towards the five normative attitudes (weighted)

	(1)	(2)	(3)	(4)	(5)	(6)
	All		Nonsupervisors		Supervisors	
	1–4 scale	Share (strongly) agree (%)	1–4 scale	Share (strongly) agree (%)	1–4 scale	Share (strongly) agree (%)
Prevent short-time work	2.88	69.28	2.90	70.86	2.77	62.02
Subsidize short-time workers	3.00	76.69	3.03	78.35	2.87	69.05
Work at home even if tasks unfulfilled (w/o children)	2.69	59.54	2.71	60.77	2.60	53.87
Work at home even if tasks unfulfilled (w/children)	3.13	82.60	3.14	82.91	3.09	81.18
Disclose vacation location	2.74	61.87	2.73	61.27	2.80	64.61
Observations	4609	4609	3599	3599	1010	1010

Source: HOPP, wave 7

To answer our first research question (RQ1)— are there norms for these topics?— we look at the respondents’ own attitudes towards the five items. In all five cases, we find that a clear majority does support the respective statement. The majority is specifically large for statements regarding the prevention of short-time work (69%), the employer’s obligation to top up wages (77%) and working from home for employees with children (83%). For these dimensions, a clear majority shares similar normative expectations. While less pronounced, a majority can also be found for working from home in case there are no children in the household present (60%) and the moral duty of employees to disclose their vacation location (62%). This result is mirrored for supervisors who are, however, slightly less supportive towards employee interests and more likely to support the employer side.

Thus far, we assumed that a social norm exists if a majority in the reference group does support the respective statement. This might not be, however, considered a particularly strong criterion. An alternative perspective is to borrow from arguments on research that specify the conditions under which we can use data collected from individuals to infer constructs on an aggregate level such as groups or organizations (Chan 1998). For example, data can be gathered from individuals on the perceived psychological climate in an organization, and these data are then aggregated to measure organizational climate (a higher level construct). In a similar vein, we can ask whether it is acceptable to aggregate individuals’ normative beliefs on a higher level and end with the reference group’s normative beliefs, which might also be called the reference group’s social norms. Theoretically rooted in the direct consensus model (Chan 1998), the so-called r_wg_ scores can be used to indicate whether shared perceptions in a group exist. In general, r_wg_ is a measure of interrater agreement and is calculated by comparing an observed variance in groups with the variance as expected from random responding, which is the variance of a null distribution or a theoretically specified distribution representing no agreement, usually a rectangular distribution (for more details and a discussion see Appendix). Higher r_wg_ scores indicate greater agreement among the respondents.

In the following, we consider the exemplary question of whether there exists evidence for separate social norms among nonsupervisory and supervisory employees. We computed the r_wg_ scores for both groups and the five items. The respective results are depicted in Table 3. The size of the r_wg_ scores can be considered a measure of the strength of the social norm, or at least of the degree to which individual beliefs are shared. From a descriptive point of view, we find first that the norms seem to be stronger among nonsupervisory employees. This might result from the fact that some participants in the supervisory group still feel as although they are somewhat “in-between” insofar as they themselves are reporting to higher-level supervisors or they consider themselves to be not more than a “primus inter pares” of their work groups. Second, the particularly low scores for the fifth statement can be taken as evidence that it is at best a norm that is in a developmental stage, although we might also conclude that evidence for its very existence is lacking. Overall, the results are in line with our estimates based on means and/or majorities for the statements.

**Table 3:** Within-group agreement indices (r_wg_) for nonsupervisory and supervisory employees

	Nonsupervisors	Supervisors
	r_wg_	r_wg_
Prevent short-time work	0.4741	0.3807
Subsidize short-time workers	0.5182	0.4855
Work at home even if tasks unfulfilled (w/o children)	0.4744	0.4271
Work at home even if tasks unfulfilled (w/children)	0.5563	0.5127
Disclose vacation location	0.0646	0.0862
Observations	3599	1010

Computations are based on a rectangular (uniform null) distribution

Source: HOPP, wave 7

Our second research question (RQ2) is whether there is a misalignment of normative attitudes between employees and supervisors. Table 2 provides descriptive evidence that supervisors lean more towards the employers’ perspective for all five attitudes. By employers’ perspective, we mean rejection of attitudes concerning obligations of employers (Items 1 to 4) and support for obligations of employees (Item 5), but that nevertheless the majority position (which is in agreement with the obligation in all five cases) is the same for employees and supervisors for all five items.

To investigate this research question further, we ran a regression on each of the items using the four-point answering scale as the dependent variable in a linear regression. These analyses (shown in Table 4) reveal that controlling for establishment attributes and respondent demographics, there is a highly significant effect for supervisors in the direction of rejecting employer obligations and supporting employee obligations for all five items. However, the effect is at most moderate and ranges in absolute size between 0.10 (for the employer’s obligation to subsidize short-time work and to allow work at home when children are present) and 0.17 (for the employer’s obligation to allow work at home when no children are present) points on the four-point scale.

**Table 4:** Linear regression of attitudes on a rich set of covariates (weighted)

	(1)	(2)	(3)	(4)	(5)
	Prevent short-time work	Subsidize short-time workers	Working at home even if tasks unfulfilled (w/o children)	Working at home even if tasks unfulfilled (w/children)	Disclose vacation location
Supervisor (0/1)	− 0.118*** (0.032)	− 0.097*** (0.030)	− 0.171*** (0.031)	− 0.098*** (0.029)	0.151*** (0.040)
Female	0.065* (0.026)	0.069** (0.025)	0.114*** (0.025)	0.092*** (0.023)	0.131*** (0.034)
Education: No vocational Training	0.000 (.)	0.000 (.)	0.000 (.)	0.000 (.)	0.000 (.)
Education: Vocational training	− 0.011 (0.082)	− 0.147* (0.073)	− 0.054 (0.080)	− 0.054 (0.074)	0.091 (0.115)
Education: Upper Secondary	− 0.229* (0.093)	− 0.284** (0.086)	− 0.012 (0.093)	− 0.040 (0.088)	0.132 (0.129)
Education: Upper Secondary + voc training	− 0.088 (0.082)	− 0.241** (0.074)	− 0.044 (0.081)	− 0.016 (0.074)	0.129 (0.116)
Education: University or FH	− 0.152 (0.080)	− 0.289*** (0.071)	− 0.018 (0.079)	− 0.000 (0.072)	0.047 (0.112)
Foreign citizen (0/1)	0.197* (0.082)	0.009 (0.081)	0.029 (0.081)	− 0.094 (0.076)	0.047 (0.110)
Age	− 0.010 (0.012)	− 0.004 (0.012)	0.004 (0.011)	− 0.012 (0.011)	− 0.010 (0.015)
Age sq./100	0.008 (0.013)	− 0.003 (0.012)	− 0.007 (0.012)	0.009 (0.011)	0.014 (0.016)
Experience (yrs)	− 0.001 (0.007)	0.004 (0.007)	− 0.016* (0.007)	− 0.003 (0.006)	− 0.004 (0.009)
Experience (yrs) sq./100	0.004 (0.014)	− 0.001 (0.014)	0.026 (0.014)	0.002 (0.013)	0.006 (0.019)
Tenure (yrs)	0.001 (0.005)	− 0.007 (0.005)	0.008 (0.005)	0.001 (0.004)	0.005 (0.007)
Tenure (yrs) sq./100	0.000 (0.019)	0.019 (0.018)	− 0.030 (0.020)	0.003 (0.016)	− 0.016 (0.026)
0–9 Employees	0.000 (.)	0.000 (.)	0.000 (.)	0.000 (.)	0.000 (.)
10–20 Employees	0.002 (0.059)	0.071 (0.056)	− 0.144* (0.059)	− 0.099 (0.055)	− 0.052 (0.074)
21–50 Employees	0.064 (0.052)	0.062 (0.051)	− 0.066 (0.052)	− 0.067 (0.049)	− 0.078 (0.067)
50 + Employees	0.022 (0.043)	0.079 (0.043)	− 0.055 (0.043)	− 0.013 (0.041)	− 0.094 (0.055)
Child under 18 in hh (0/1)	− 0.002 (0.028)	0.010 (0.027)	− 0.066* (0.028)	0.073** (0.026)	0.137*** (0.037)
Daily pay in €100 (2019)	0.032 (0.026)	0.040 (0.024)	− 0.017 (0.024)	− 0.037 (0.023)	− 0.026 (0.033)
Short-time allowance (0/1)	− 0.263*** (0.047)	0.019 (0.043)	− 0.037 (0.045)	− 0.059 (0.042)	− 0.176** (0.058)
Possibility for working at home (0/1)	− 0.015 (0.028)	− 0.008 (0.027)	0.181*** (0.028)	0.191*** (0.025)	0.034 (0.037)
Constant	3.148*** (0.224)	3.220*** (0.221)	2.843*** (0.214)	3.470*** (0.200)	2.824*** (0.295)
Observations	4609	4609	4609	4609	4609
*R*^2^	0.022	0.018	0.041	0.040	0.014

Dependent var: Individual attitude towards the item

^*^ *p* < 0.05, ^**^ *p* < 0.01, ^***^ *p* < 0.001

Source: HOPP, wave 7

The finding that there are significant differences between employees and supervisors in the expected direction might point towards the potential to produce tensions and conflicts in ERs since employees will expect the supervisor to adhere to the respective norm and vice versa. However, one must conclude that this misalignment is rather small, and for all five attitudes, the majority position is the same in both groups. In principle, supervisors hold the same normative attitudes as employees, which will strongly limit potential conflicts over the norm’s role.

Turning to the control variables in the regression displayed in Table 4, structural variables, specifically establishment size or education, do not influence the extent of agreement with the statements. However, we find significant correlations supporting the role of self-interest. There are significant but small positive effects for children in the household and for respondents with the possibility of working at home on attitudes regarding the right to work at home. Moreover, women seem to be slightly more supportive regarding all normative statements, which could be explained by the fact that women were affected more by the pandemic’s labour market consequences than men (Hammerschmid et al. 2020). The absence of further significant correlations highlights that the attitudes in question do not vary by respondents’ age or education or between employees in establishments of different sizes.

To sum up the results for RQ1 and RQ2 at this point, we find empirical evidence that nonsupervisory employees and superiors share similar normative attitudes towards our five items. The share of supporters seems to be higher for the three items that deal with a strong need of employees, that is, income in the case of short-time work and the reconciliation of family and work under pandemic conditions (work from home with children). The lowest percentage of agreement can be found for employers’ wish for private information. There are different possible explanations for this fact. For example, this finding is in accordance with the assumption that norms to protect employees are more prominent than norms covering the need of employers. Another possibility is that it is more difficult to establish a norm for handling private information due to the short time during which this problem was important. Moreover, disclosing the vacation location may protect coworkers, which could produce normative pressure from the staff.

Turning to the third research question (RQ3), we explore whether the observed extent of agreement of individual normative beliefs in our sample differs from the extent of agreement concerning respondents’ estimates for the majority in the labour force. This allows us to answer the question of whether there is pluralistic ignorance concerning the five normative attitudes. Table 5 shows the means for the full Likert scale (1–4) and the differences between means for respondents’ own attitudes and the estimated attitude for the majority of employees.

**Table 5:** Differences between respondents’ attitudes and assessed majority attitudes, 1–4 scale (weighted)

	(1)	(2)	(3)	(4)	(5)	(6)	(7)	(8)	(9)
	All			Nonsupervisors			Supervisors		
	Majority	Respondent	Diff.	Maj.	Resp.	Diff.	Maj.	Resp.	Diff.
Prevent short-time work	3.23	2.88	0.35***	3.24	2.90	0.33***	3.22	2.77	0.45***
Subsidize short-time workers	3.30	3.00	0.30***	3.29	3.03	0.26***	3.34	2.87	0.46***
Working at home even if tasks unfulfilled (w/o child.)	2.79	2.69	0.10***	2.77	2.71	0.06***	2.89	2.60	0.29***
Working at home even if tasks unfulfilled (w/child.)	3.18	3.13	0.05***	3.18	3.14	0.04*	3.20	3.09	0.11***
Disclose vaca-tion location	2.19	2.74	− 0.55***	2.20	2.73	− 0.54***	2.18	2.80	− 0.62***
Observations	4609	4609	4609	3599	3599	3599	1010	1010	1010

*p < 0.05, **p < 0.01, ***p < 0.001, two tailed t-tests were used to test the statistical significance of the difference

Source: HOPP, wave 7

For all attitudes, we observe a statistically significant difference between the mean in our sample and the respondents’ estimate for the majority. For the first four items, we see that people overestimate normative attitudes in the population. Hence, we find a general tendency towards pluralistic ignorance for these normative attitudes. The results of the supervisors, which show somewhat higher differences, can be explained by their lower level of support of the normative attitudes in their group. Interestingly, the supervisors’ estimates of the majority do not differ from the employees’ estimates. This means that although the supervisors have a different opinion concerning these attitudes, they have the same belief about the attitudes of the majority, which is an important indicator that the social norms behind these attitudes are acknowledged by both groups. Supervisors know about these norms; thus, it is plausible to assume that they will take potential employees’ reactions to norm deviance into account.

However, there are also noticeable differences. For both items on working from home, we see statistically significant but only small effects (0.04 and 0.06, respectively). The respondents’ estimates of the majority’s attitudes are not far from the mark and are considerably lower than for the other three items. This is in line with our assumption that extensive public discourse on a topic will improve assessments and reduce the probability and extent of pluralistic ignorance. We have already argued and have shown in Appendix that working from home was a heavily discussed topic among general the public and in politics. This allowed people to learn more about the opinions of others and thus may have improved the estimate of the majority’s attitudes.

Finally, we find that the normative attitude on the disclosure of vacation location seems to be a special case because the respondents’ attitudes are contrary to the estimation of the majority’s opinions: whereas 62 percent agree that the employer should get this information, only 34 percent think that this is the opinion of the majority. There are two possible interpretations for this finding. First, it could be a specifically distinctive case of pluralistic ignorance if people think that employers should know about vacation locations, but they do not disclose this information because they expect to be sanctioned by the majority of employees. Second, people assume that they have different attitudes than the majority, but they do not care because they do not expect to be sanctioned. The latter would mean that there is not truly a shared social norm on this subject. Without any information on expected sanctions, which would be a crucial requirement for the existence of a valid social norm (Bicchieri 2006, 2017), we are unable to determine which option is valid.

## Conclusions

In this paper, we examined normative attitudes towards a selected set of behaviours that became a particular focus of public attention in the context of the COVID-19 pandemic. To our knowledge, this is the first study taking up this topic. Specifically, we looked at attitudes towards the assignment and compensation of short-time work and work from home by employers as well as attitudes towards the sharing of private information on employees’ travel behaviour with the employer.

First, we asked whether consistent patterns exist for a majority of respondents, indicating that there are social norms on this subject. We found that a majority of employees and supervisors agreed with the normative statements, indicating a shared understanding of how to behave in the respective situation. Second, we explored whether and to what extent there is a misalignment of normative attitudes between employees and supervisors. If supervisors support a norm less than employees do, this can be a source of conflict in the employment relationship (Görges and Nosenzo 2020). We found evidence for misalignment for all five attitudes investigated, since supervisors reported significantly less support for the employees’ position. Third, we examined differences between respondents’ average attitudes on the one hand and the attitudes they expect to exist in the population on the other. This allows investigating whether people overestimate the support for a social norm, which is also known as pluralistic ignorance in the literature (Shamir and Shamir 1997). We found a tendency towards pluralistic ignorance for all items, albeit the effect for the two items on working from home was rather small. For the two items on short-time work, however, people overestimated the support for the norm with the possible consequence of contributing to the upholding of the norm even if they do not support the norm themselves. Finally, the results for the disclosure of vacation locations revealed that it is not clear whether there is a social norm on the topic. These results on four exemplary norms also shed light on the more general role of norms for employment relationships for regulating behaviour and conflicts. Obviously, employees with and without supervisory functions share those norms – even if those norms lead to costs mostly for the employee or the employer. This is an important requirement for effective norms regulating behaviour in problematic situations.

Although we think that we can contribute to the question of how social norms influence the employment relationship and, thus, labour markets, there are some caveats. First, we measured only attitudes; hence, we do not know to what extent people act on issues. Second, we measured attitudes at one point in time, which restricts our analysis to cross-sectional models. Hence, we are not able to answer the question of how the pandemic changed attitudes and norms over time and whether this led to pluralistic ignorance. Third, the measurement of pluralistic ignorance could be biased if individuals with a specific positive attitude towards the norms have a higher probability of participating in the survey. Specifically, one could argue that respondents agreeing to linkage of the survey to the administrative data are more law-abiding and thus more prone to norms in general. This would lead to an overestimation of the difference between the sample and the estimation for the population by the respondents. However, although the participation rate was rather low in the HOPP survey, we deem this kind of bias unlikely: the linkage consent rate is not higher than in existing comparable surveys. Moreover, weighting generated from high-quality administrative data should further reduce potentially remaining biases. Finally, an interesting question for future research is how people react when social norms are violated. How, for example, would an employee react if the employer denied the possibility of working at home? One possibility would be to sanction the employer; possible retaliatory measures might be, for example, to protest publicly in the firm, to reduce work performance or to resign and change employers (Hirschman 1970).

However, despite these limitations, we can shed light on the question of how normative attitudes and social norms influence the employment relationship. People obviously not only form beliefs about how employees and supervisors should behave but also about how the majority thinks about particular topics. This is the very basis of social norms and the prerequisite for regulating behaviour in employment relationships by “soft” sanctions such as esteem, displeasure or publicly displayed indignation. We showed that supervisors know about these attitudes and norms, and although they do not share them to exactly the same extent as employees, it is plausible to assume that they take these norms into account when making personnel decisions.

## Data Availability

The HOPP-data used in this paper are available via the Research Data Centre (FDZ) of the Federal Employment Agency at the Institute for Employment Research. Due to data protection regulations, the administrative records on STW are currently—to the best of our knowledge—only available within the Institute for Employment Research.

## Appendix

### Discussion of topics during the pandemic

This graph shows the Google Trends search intensity for the terms “Home office corona”, “Kurzarbeitergeld corona” (*Short-term allowance corona)* and “Urlaubsort Arbeitgeber” (*Vacation destination employer).* 100 indicates the highest search frequency for any of the terms during the observed period; search intensities are measured relative to this peak.

### German questionnaire for the five normative attitudes

[NA1000] Nun geht es darum, wie Arbeitgeber mit den Folgen der Corona-Krise umgehen: Inwieweit stimmen Sie persönlich den folgenden Aussagen zu?

- Arbeitgeber haben die moralische Pflicht, Kurzarbeit ihrer Arbeitnehmer zu vermeiden solange im Betrieb noch finanzielle Rücklagen vorhanden sind.
- Arbeitgeber haben die moralische Pflicht, das Kurzarbeitergeld aufzustocken, solange im Betrieb noch finanzielle Rücklagen vorhanden sind.
- Arbeitnehmer ohne Kinder sollten auch dann zu Hause arbeiten können, wenn sie dort nicht alle Aufgaben erledigen können.
- Arbeitnehmer mit Kindern sollten auch dann zu Hause arbeiten können, wenn sie dort nicht alle Aufgaben erledigen können.
- Arbeitnehmer haben in Zeiten der Corona-Krise die moralische Pflicht, ihrem Arbeitgeber mitzuteilen, wo sie sich im Urlaub aufgehalten haben.
- Stimme voll und ganz zu.
- Stimme eher zu.
- Stimme eher nicht zu.
- Stimme überhaupt nicht zu.

[NA2000] Nicht immer entspricht die eigene Meinung der der Mehrheit. Was glauben Sie, dass die Mehrheit der Erwerbstätigen für die folgenden Aussagen in Bezug auf den Umgang eines Arbeitgebers oder eines Arbeitnehmers mit den Folgen der Corona-Krise ankreuzen wird?

- Arbeitgeber haben die moralische Pflicht, Kurzarbeit ihrer Arbeitnehmer zu vermeiden solange im Betrieb noch finanzielle Rücklagen vorhanden sind.
- Arbeitgeber haben die moralische Pflicht, das Kurzarbeitergeld aufzustocken, solange im Betrieb noch finanzielle Rücklagen vorhanden sind.
- Arbeitnehmer ohne Kinder sollten auch dann zu Hause arbeiten können, wenn sie dort nicht alle Aufgaben erledigen können.
- Arbeitnehmer mit Kindern sollten auch dann zu Hause arbeiten können, wenn sie dort nicht alle Aufgaben erledigen können.
- Arbeitnehmer haben in Zeiten der Corona-Krise die moralische Pflicht, ihrem Arbeitgeber mitzuteilen, wo sie sich im Urlaub aufgehalten haben.

Die Mehrheit der Erwerbstätigen wird auf die folgenden Fragen antworten…

1. Stimme voll und ganz zu.
2. Stimme eher zu.
3. Stimme eher nicht zu.
4. Stimme überhaupt nicht zu.

### Definition and discussion of the r_wg_ score

As already pointed out, the r_wg_ score provides a variance-based measure for interrater agreement and is calculated by comparing an observed variance in groups either with the variance as expected from random responding, which is the variance of a null distribution, or a theoretically specified distribution representing no agreement, usually a rectangular distribution. The formulas are presented in various sources (Cohen et al. 2001):

${\text{r}}_{{{\text{wg}}}} \, = \,{1}{-}({\text{s}}_{{\text{x}}}^{{2}} / \, \sigma^{{2}} )$ where s_x_^2^ is the observed variance in ratings of the item and σ^2^ is the variance of the null distribution. Higher r_wg_ scores indicate greater agreement. The most common way to specify the null distribution is

$\sigma^{{2}} \, = \,\left( {{\text{A}}^{{2}} {-}{1}} \right)/{12}$ with A representing the number of categories (in the current case = 4).

It should be noted that there is considerable debate on the interpretation of the size of r_wg_ scores as well as the appropriateness of the kind of null distribution to use. For example, some scholars have suggested that 0.70 should be taken as a threshold for justifying aggregation (e.g., Lance et al. 2006), although Smith-Crowe et al. (2014) show that in case that the number of judges increases, the threshold for justifying aggregation can and should decrease to a considerable extent. As an alternative, testing of the statistical significance of r_wg_ by means of Monte Carlo simulations has been suggested (Cohen et al. 2009). However, as seen in Smith-Crowe et al. (2014), if the number of judges in the groups exceeds 100 (as is clearly the case in our samples), a test of the significance of r_wg_ becomes increasingly less informative. Finally, a rectangular distribution might not seem to be the most appropriate assumption because, per definition, in the case of a strong social norm, the distribution of answers within a reference group should be skewed. However, for single items and four categories, even under the assumption of heavy skewness (e.g., 00/0.05/0.40/0.55), the critical value for n = 100 judges is 0.19. Thus, to the best of our knowledge, in the case of large group sizes, we should also take the absolute size of r_wg_ into account.

## Footnotes

1 The high proportion of individuals who give the linkage consent is consistent with other IAB-surveys that allow for record linkage, e.g. the Panel Study Labour Market and Social Security (PASS) with a 94% consent rate (Trappmann et al. 2019).

2 Note that daily pay is censored at the social security contribution assessment ceiling, around the 85^th^ percentile of the earnings distribution. This leads to an underestimation of daily pay in our effective sample.
